# Ocular surface status in glaucoma and ocular hypertension patients with existing corneal disorders switched from latanoprost 0.005% to tafluprost 0.0015%: comparison of two prostaglandin analogues with different concentrations of benzalkonium chloride

**DOI:** 10.1111/ceo.13329

**Published:** 2018-06-26

**Authors:** Tina T Wong, Tin Aung, Ching Lin Ho

**Affiliations:** ^1^ Singapore National Eye Centre (SNEC) Singapore Singapore; ^2^ Singapore Eye Research Institute (SERI) Singapore Singapore; ^3^ Duke‐NUS Graduate Medical School Singapore Singapore; ^4^ Yong Loo Lin School of Medicine National University of Singapore Singapore Singapore

**Keywords:** corneal disease, latanoprost, ocular hypertension, primary open‐angle glaucoma, tafluprost ophthalmic solution

## Abstract

**Importance:**

Glaucoma treatment has often been associated with adverse side‐effects from preservatives that are included in the used eye drops.

**Background:**

To evaluate changes in the ocular surface and the presence of prostaglandin‐induced corneal disorders after being switched from latanoprost 0.005% to low preservative tafluprost 0.0015% ophthalmic solution.

**Design:**

Single centre, prospective study.

**Participants:**

Patients with primary open‐angle glaucoma or ocular hypertension that had received treatment with once daily latanoprost 0.005% ophthalmic solution for control of intraocular pressure (IOP) for 3 months, with a score of above 1 on the National Eye Institute (NEI) ocular surface staining scale.

**Methods:**

Following the ≥3 month latanoprost treatment period, patients were switched to once daily low preservative tafluprost 0.0015% ophthalmic solution. Patients were followed for a minimum of 3 months.

**Main Outcome Measures:**

Ocular surface changes were assessed by fluorescein staining score (NEI scale). Additional evaluations included tear break‐up time, hyperaemia score, subjective symptoms, changes in intraocular pressure and presence of adverse reactions.

**Results:**

Out of 59 patients enrolled, 51 were included in the final analysis. Fluorescein staining scores at baseline, prior to treatment switch, were 6.9 ± 3.1 and 3.3 ± 2.7 at the end of the study period (change in scores was −3.6 ± 2.2 [*P* < 0.001]). At last follow‐up, significant improvements were observed in tear break‐up time, hyperaemia score and subjective symptoms (all *P* < 0.05).

**Conclusions and Relevance:**

The clinical signs of ocular surface disease and subjective symptoms of dry eyes improved following the switch to low preservative tafluprost and demonstrated comparable IOP lowering effectiveness.

## Introduction

Glaucoma is a progressive disease affecting the optic nerve and is the second leading cause of blindness worldwide.^1^ Although the aetiology of glaucoma is multifactorial, the reduction of intraocular pressure (IOP) is currently the only evidence‐based treatment strategy for slowing its progression.^2^ In order to reduce IOP to acceptable levels in primary open‐angle glaucoma (POAG) and ocular hypertension (OH), topical hypotensive medications remain the standard for initial treatment.

Prostaglandin analogues are currently considered the first‐line therapy as they have demonstrated potency and efficacy in lowering IOP with an acceptable side‐effect profile. The most common prostaglandin analogues are latanoprost, bimatoprost and travoprost, which have shown similar IOP‐lowering efficacies.^3^

Although effective, most of these currently available medications contain preservatives such as benzalkonium chloride (BAC), a commonly used quaternary ammonium cationic surface‐acting agent that dissolves bacterial walls and membranes like a detergent.^4^ Bimatoprost 0.01% and latanoprost 0.005% each contain 0.02% BAC and travoprost 0.004% contains 0.015% BAC.

These topical medications are not curative and patients with POAG or OH will require consistent and prolonged treatment in order to maintain normal IOP. Unfortunately, experimental and clinical studies have shown that chronic exposure to BAC has been associated with ocular surface changes, such as modification and/or disruption of the lipid layer of the tear film and various ocular surface disorders which can include hyperaemia, punctate keratitis, burning or foreign body sensation and dry eyes.^5^, ^6^, ^7^

In 2002, a prospective long‐term epidemiological survey of 4107 glaucoma patients was conducted to assess the effects of preserved and preservative‐free eyedrops on ocular symptoms and conjunctival, corneal and palpebral signs in clinical practice.^6^ The study found that the symptoms reported by patients, which included discomfort, burning, stinging and foreign body sensations, dry eye, tearing and itchy eyelids, were significantly more prevalent in patients using topical medication containing preservatives, when compared to those receiving preservative‐free treatment.^6^ Furthermore, a study comparing the cytotoxicity of anti‐glaucoma drugs with different BAC concentrations suggested that the cytotoxicity of eyedrops is dependent on BAC. Amongst all eyedrops tested, only those containing latanoprost or tafluprost showed a reduction in the cytotoxicity of BAC.^8^

Due to the necessity of long‐term treatment for these patients and the high incidence of hypersensitivity to the therapy or the preservatives it contains, this has become a significant concern for ophthalmologists, and the ophthalmic industry. Therefore, in an effort to reduce ocular symptoms and avoid the corneal damage and tear film disruption associated with chronic BAC exposure, preservative‐free or low preservative‐containing formulations of existing anti‐glaucoma eye drops have been developed.

Tafluprost 0.0015% (Santen Inc., Osaka, Japan), a low preservative prostaglandin analogue has demonstrated similar IOP reduction over a 24‐h period when compared to branded latanoprost.^9^ In terms of preservative, commercially available tafluprost ophthalmic solution contains 0.001% BAC, which is 20 times lower concentration than latanoprost ophthalmic solution. Tafluprost 0.0015% is an ophthalmic solution in which BAC concentration is recently reduced. Additionally, reduced prevalence of superficial punctate keratopathy (SPK) has been shown in a clinical comparison of tafluprost to latanoprost.^10^ In this study, we evaluated changes in the ocular surface status and IOP lowering effects of patients with POAG or OH and the presence of existing prostaglandin‐induced corneal disorders after being switched from latanoprost 0.005% to tafluprost 0.0015% ophthalmic solution.

## Methods

This was a prospective, single‐centre study. Fifty‐nine patients from Singapore National Eye Centre with POAG or (OH) with concurrent corneal disorders due to latanoprost treatment were enrolled in the study. Corneal disorders due to prostaglandin usage were defined as a score above 1 on the National Eye Institute (NEI) ocular surface staining scores, indicative of ocular surface damage.

The study was designed to closely mimic actual clinical practice with a primary objective to investigate the effect on the cornea in POAG or OH patients, when switched from latanoprost 0.005% ophthalmic solution (Pfizer, New York, NY, USA) to low preservative tafluprost 0.0015% ophthalmic solution.

The primary endpoint was the change in the fluorescein staining score (NEI scale) at the end of the study period (≥month 3). Secondary endpoints included tear break‐up time (TBUT), hyperaemia score, subjective symptoms, IOP and adverse drug reactions (ADRs).

Specific inclusion criteria included patients over 21 years of age, an IOP not exceeding 22 mmHg at baseline and managed with latanoprost 0.005% monotherapy for at least 3 months prior to baseline. All patients also had to have corneal disorders due to latanoprost usage, defined as an NEI score > 1 in at least one eye. Patients with severe visual field disorders, dry eyes, ocular allergies, active infection or inflammation of the eye, those taking systemic or ophthalmic steroids or other anti‐glaucoma agents, patients using contact lenses or those who had undergone ocular surgeries within the previous 6 months were excluded from the study.

Patients were enrolled between February 2014 and February 2015 and provided written informed consent. Ethical approval was provided by the institution review board. At enrolment, all patients had been receiving once‐daily latanoprost 0.005% for 3 months or more. The baseline visit (Day 0) represented the day the patients stopped treatment with latanoprost and were switched to once‐daily low preservative tafluprost 0.0015% ophthalmic solution. Treatment was administered as indicated per the package insert, one drop at a time, once daily. Patients remained on tafluprost treatment for a period of at least 3 months.

Study patients attended two follow‐up visits at 1 month and 3 months following treatment switch. At each of these visits, patients underwent IOP measurement three times using Goldmann applanation tonometry, and evaluation of ocular surface disease by TBUT measurement and calculation of SPK and hyperaemia scores.

In the NEI/I method, the cornea is divided into five sections: central; superior; temporal; nasal and inferior. The density of fluorescein punctate staining in each section was scored as follows: 0 = none; 1 = sparse; 2 = dense and 3 = coalesced. For conjunctival hyperaemia, a 5‐point grading scale (0 = none; 1 = mild; 2 = moderate; 3 = severe and 4 = very severe) was used. The cornea or tear fluid was stained with fluorescein test paper, and SPK and TBUT observations were made using a slit‐lamp microscope. Corneal staining scores and assessment of conjunctival hyperaemia were determined using grading scales, and single grading tables with reference photographs. If both eyes were eligible for enrolment in the study, the eye with the higher fluorescein staining score was selected as the study eye (if the scores were the same, the right eye was selected).

Additionally, patients completed a subjective questionnaire related to their ocular surface disease symptoms including: eye discharge, eye pain, foreign body sensation, irritation (burning/stinging), itching, photophobia and tearing. Details regarding patient compliance to treatment and presence of any adverse events were also recorded at each visit.

We calculated that a sample size of 56 would be expected to have 80% power to detect a difference in means of 1.0 between baseline and visit 2 in the fluorescein staining score, assuming a standard deviation of differences of 2.6, using a paired *t*‐test with a 0.05 two‐sided significance level. Considering the possibility of study discontinuation of treatment for some patients, estimated at approximately 6%, a sample size of 60 was planned.

Last Observation Carried Forward (LOCF) imputation was implemented for the analysis at end of the study period. The Full Analysis Set (FAS), which included all subjects who had corneal disorders due to prostaglandin (PG) usage, had instilled the study drug at least once and provided at least one post‐baseline corneal fluorescein staining score, was used for both safety analysis and efficacy analysis.

Change in the fluorescein staining score (NEI scale) at the end of the study period (visit 2/month 3) was determined and differences were calculated by paired t‐test. *P* values were considered statistically significant if less than 0.05. Secondary endpoints including TBUT, hyperaemia score and IOP changes from baseline were summarized by study time point and means, standard deviations (SD) and *P* values were calculated. Adverse drug reactions and subjective symptoms were summarized by frequency for each time point.

## Results

### Patient disposition, demographics and baseline characteristics

Of the 59 patients who gave informed consent and were administered with study drug, 47 (79.7%) completed the study, while seven (11.9%) discontinued early due to adverse events (i.e. eye redness, feeling of discomfort and foreign body sensation), two (3.4%) were lost to follow up and 3 (5.1%) discontinued due to other reasons (i.e. prolonged period between follow‐up visits, insufficient eyedrop to last for next visit period). Therefore, the full analysis set (FAS) included 51 patients. The average (mean ± SD) age was 66.6 ± 11.7 years with a range of 39–91 years and 56.9% were female. The baseline IOP (mean ± SD) was 14.4 ± 3.2 mmHg (Table 1).

**Table 1:** Demographics and baseline characteristics, full analysis set (FAS)

		*n* (%)
All patients	*N*	51
Diagnosis	Primary open‐angle glaucoma	40 (78.4)
	Ocular hypertension	11 (21.6)
Sex	Male	22 (43.1)
	Female	29 (56.9)
Age (years)	Minimum–maximum	39–91
	Mean ± SD	66.6 ± 11.7
Age group	<65 years	24 (47.1)
	≥65 years	27 (52.9)
Baseline IOP (mmHg)	Minimum–maximum	7.5–22.0
	Mean ± SD	14.4 ± 3.2

IOP, intraocular pressure.

### Primary endpoint

Fluorescein staining scores (mean ± SD) at baseline and end of the study period were 6.9 ± 3.1 and 3.3 ± 2.7, respectively. Change in scores (mean ± SD) at end of the study period were −3.6 ± 2.2 (*P* < 0.001, [95% CI ‐4.5, −2.7]). Changes in the fluorescein staining score are noted for each of five corneal areas: central; superior; temporal; nasal and inferior; and clearing percentage at each visit is summarized in Table 2.

**Table 2:** Results of fluorescein staining scores (NEI scale), at baseline, month 1 and end of study period (≥3 months). *N* = 51

Corneal area	Visit	Score	Change in scores			Clearing	
		Mean ± SD	Mean ± SD	95%CI	*P* value	*N*	*n* (%)
A: Central	Baseline	0.7 ± 0.8	–	–	–	30	4 (13.3)
	Month 1	0.3 ± 0.7	−0.4 ± 0.8	–	0.003	30	19 (63.3)
	End of study period	0.2 ± 0.4	−0.5 ± 0.8	(−0.7,‐0.2)	<0.001	30	19 (63.3)
B: Superior	Baseline	0.8 ± 1.1	–	–	–	33	8 (24.2)
	Month 1	0.3 ± 0.8	−0.5 ± 1.3	–	0.010	33	23 (69.7)
	End of study period	0.3 ± 0.6	−0.5 ± 1.1	(−0.8,‐0.2)	0.001	33	22 (66.7)
C: Temporal	Baseline	1.3 ± 0.9	–	–	–	48	6 (12.5)
	Month 1	0.4 ± 0.5	−0.9 ± 0.9	–	<0.001	48	30 (62.5)
	End of study period	0.6 ± 0.7	−0.7 ± 1.0	(−1.0,‐0.4)	<0.001	48	21 (43.8)
D: Nasal	Baseline	1.6 ± 0.9	–	–	–	50	4 (8.0)
	Month 1	0.7 ± 0.7	−0.9 ± 1.0	–	<0.001	50	23 (46.0)
	End of study period	0.8 ± 0.8	−0.8 ± 1.3	(−1.1,‐0.4)	<0.001	50	19 (38.0)
E: Inferior	Baseline	2.6 ± 0.9	–	–	–	51	0 (0.0)
	Month 1	1.5 ± 1.0	−1.1 ± 1.2	–	<0.001	51	5 (9.8)
	End of study period	1.4 ± 1.0	−1.2 ± 1.2	(−1.5,‐0.8)	<0.001	51	6 (11.8)
Total (entire corneal area)	Baseline	6.9 ± 3.1	–	–	–	51	0 (0.0)
	Month 1	3.2 ± 2.5	−3.8 ± 3.3	–	<0.001	51	3 (5.9)
	End of study period	3.3 ± 2.7	−3.6 ± 3.2	(−4.5,−2.7)	<0.001	51	6 (11.8)

When compared to the entire cornea, all individual areas also showed significant decreases at both month 1 and end of the study period compared with baseline (all *P* < 0.05). Clearing percentage in the entire cornea at baseline, month 1 and end of the study period was 0%, 5.9% and 11.8%, respectively. At the end of the study period, clearing in the individual corneal areas were: central 63.3%, superior 66.7%, temporal 43.8%, nasal 38.0% and inferior 11.8%; notably much higher than at baseline.

### Secondary endpoints

Analysis results for secondary endpoints, which included TBUT, hyperaemia score, subjective symptoms and IOP are provided in Table 3.

**Table 3:** Results of secondary endpoints at baseline, month 1 and end of study period (≥3 months). *N* = 51

Variable	Visit	*N* = 51	Value	Change in value	
			Mean ± SD	Mean ± SD	*P* value
TBUT (second)	Baseline		3.0 ± 1.8	–	–
	Month 1		4.1 ± 2.9	1.1 ± 2.5	0.003
	End of study period		3.7 ± 2.5	0.7 ± 2.1	0.017
Hyperaemia score					
Bulbar conjunctiva	Baseline		1.7 ± 0.5	–	–
	Month 1		1.6 ± 0.6	−0.2 ± 0.6	0.028
	End of study period		1.5 ± 0.5	−0.3 ± 0.5	0.001
Palpebral conjunctiva	Baseline		1.8 ± 0.6	–	–
	Month 1		1.8 ± 0.6	−0.1 ± 0.6	0.497
	End of study period		1.5 ± 0.5	−0.3 ± 0.6	0.001
Subjective symptoms (OSD score)					
Eye discharge	Baseline		0.2 ± 0.4	–	–
	Month 1		0.2 ± 0.4	0.0 ± 0.5	0.799
	End of study period		0.2 ± 0.4	−0.1 ± 0.5	0.290
Eye pain	Baseline		0.1 ± 0.4	–	–
	Month 1		0.0 ± 0.2	−0.1 ± 0.4	0.261
	End of study period		0.0 ± 0.0	−0.1 ± 0.4	0.058
Foreign body sensation	Baseline		0.4 ± 0.6	–	–
	Month 1		0.2 ± 0.4	−0.1 ± 0.7	0.164
	End of study period		0.0 ± 0.2	−0.3 ± 0.5	<0.001
Irritation (burning/stinging)	Baseline		0.3 ± 0.7	–	–
	Month 1		0.2 ± 0.4	−0.2 ± 0.8	0.146
	End of study period		0.0 ± 0.1	−0.3 ± 0.7	0.006
Itching	Baseline		0.5 ± 0.8	–	–
	Month 1		0.2 ± 0.4	−0.3 ± 0.8	0.010
	End of study period		0.1 ± 0.3	−0.4 ± 0.8	<0.001
Photophobia	Baseline		0.4 ± 0.6	–	–
	Month 1		0.1 ± 0.3	−0.3 ± 0.7	0.011
	End of study period		0.2 ± 0.4	−0.2 ± 0.6	0.009
Tearing	Baseline		0.2 ± 0.5	–	–
	Month 1		0.2 ± 0.4	0.0 ± 0.6	1.000
	End of study period		0.3 ± 0.5	0.0 ± 0.5	0.799
Total score	Baseline		2.2 ± 2.7	–	–
	Month 1		1.2 ± 1.3	−0.9 ± 2.8	0.019
	End of study period		0.7 ± 1.1	−1.4 ± 2.3	<0.001
IOP (mmHg)	Baseline		14.4 ± 3.2	–	–
	Month 1		14.2 ± 3.5	−0.2 ± 2.7	0.645
	End of study period		14.3 ± 2.8	−0.1 ± 3.1	0.874

IOP, intraocular pressure; OSD, ocular surface disease; TBUT, tear break‐up time.

Changes in TBUT (mean ± SD) at month 1 and month 3 were 1.1 ± 2.5 s (*P* = 0.003) and 0.7 ± 2.1 s (*P* = 0.017), respectively. Significant extension of TBUT was observed compared with baseline.

At month 1 and last follow up (≥month 3), changes in hyperaemia score were −0.2 ± 0.6 (*P* = 0.028) and −0.3 ± 0.5 (*P* = 0.001) for bulbar conjunctiva, respectively, and −0.1 ± 0.6 (*P* = 0.497) and −0.3 ± 0.6 (*P* = 0.001) for palpebral conjunctiva, respectively. Significant decreases in hyperaemia score for both bulbar conjunctiva and palpebral conjunctiva were observed at the last follow‐up visit.

An ocular surface disease (OSD) questionnaire evaluated the subjective symptoms experienced by patients and results are listed in Table 3. At the end of the follow‐up period, patients scored symptoms of foreign body sensation, irritation (burning/stinging), itching and photophobia significantly lower than they had at baseline (*P* < 0.05). With regards to the total OSD score, 30 (58.8%) patients reported ‘decreased (pre > post)’, 18 (35.3%) ‘unchanged (pre = post)’ and 3 (5.9%) ‘increased (pre < post)’ when comparing their baseline symptoms to the last follow‐up visit.

Changes in IOP (mean ± SD) at month 1 and month 3 were −0.2 ± 2.7 mmHg (*P* = 0.645) and −0.1 ± 3.1 mmHg (*P* = 0.874), respectively. No significant difference was observed in IOP compared with baseline. Stable IOP lowering action was observed for the duration of the study period.

### Adverse drug reactions

A total of four ocular ADRs (conjunctival haemorrhage, eye discharge, eye pain, foreign body sensation in eyes, each 1.7% [1/59]) and one systemic ADR (dizziness, 1.7% [1/59]) were reported in three out of 59 (5.1%) patients during the study period. One (1.7%) patient discontinued the study due to systemic dizziness. All events reported during the study period were mild in severity and consistent with existing ADR information in the package insert. No safety issues were identified.

## Discussion

The primary goal of glaucoma treatment is to reduce the raised IOP to acceptable levels. In the current study, our patients with either POAG or OH had adequate control of IOP with latanoprost therapy; however they were experiencing clinical signs of ocular surface disease, as is often associated with prolonged use of BAC containing prostaglandin analogues. We investigated whether switching these patients from BAC preservative‐rich latanoprost to low preservative tafluprost could maintain IOP control and reduce ocular surface symptoms.

No statistically significant differences in IOP were found following treatment switch, which is consistent with previous studies demonstrating similar IOP lowering efficacies.^9^, ^10^

BAC, the preservative found in most common prostaglandin analogue ophthalmic solutions, has been shown to increase the prevalence of ocular surface changes.^5^, ^6^, ^7^ These changes include modification and/or disruption of the lipid layer of the tear film and can lead to various ocular surface disorders which can include hyperaemia, punctate keratitis, burning or foreign body sensation, and dry eye.^5^, ^6^, ^7^

Since glaucoma is a chronic disease, treatment must be consistently administered over long periods of time, with the majority of patients administering these drops for several decades. This extended treatment and prolonged exposure to BAC increases a patient's susceptibility to ocular surface adverse reactions.^4^, ^11^ Thus, by reducing the use of BAC and other preservatives from the treatment, as in our case with the use of low preservative tafluprost, these negative side effects on ocular surface can be minimized.

Additionally, often as glaucoma progresses, these patients may receive several different types of eye drops during the course of their condition to maintain acceptable IOP and the addition of further treatment regimens may lead to increased development or severity of ocular surface disorders.^12^

Despite the treatment regimen being once daily for the topical medications used in this study, studies have demonstrated that patients have challenges adhering to the prescribed dosing. A study conducted at the Singapore National Eye Centre reviewed the pharmacy dispensing records for patients prescribed monotherapy for IOP management over a 3‐year period. They found that only 22.5% of patients still continued with their IOP lowering regimen at 1 year, which fell further, to 11.5% at 3 years.^13^

Since glaucoma requires continued treatment, increased adherence to a treatment regimen is an important factor for a successful outcome. Objective signs (such as corneal epithelial disorder and conjunctival hyperaemia) as well as subjective symptoms (such as foreign body sensation and irritation) are key factors in improving adherence, and thus the overall disease management.

In our study, patients reported a reduction in symptoms related to ocular surface discomforts. We hypothesise that by reducing patient discomfort, adherence to the prescribed regimen may be improved and patients may remain in therapy rather than opting to discontinue, thus improving treatment outcomes.

Fluorescein staining scores, using the NEI scale, were calculated in order to quantify the changes or improvements on corneal disorders in POAG and OH patients who switched from latanoprost to tafluprost ophthalmic solution. The study results showed that the fluorescein staining scores were significantly decreased at 1 month after switch and the effect continued up to month 3. All individual areas of the cornea demonstrated similar improvement during the treatment period. Clearing percentage of fluorescein staining in the central part of the cornea, highly associated with visual function, was 63.3% at month 3, much higher than 13.3% noted at baseline. Significant improvements on TBUT and hyperaemia (both bulbar conjunctiva and palpebral conjunctiva) were also observed in this study.

Results of this study suggest that, while tafluprost ophthalmic solution has a stable IOP lowering effect equal to latanoprost ophthalmic solution, a few adverse reactions were reported during the follow‐up period that resulted in withdrawal from the study. Furthermore, in addition to the objective clinical signs, study results also demonstrated improvements in subjective symptoms, such as foreign body sensation, irritation, itching and photophobia.

We acknowledge that this study has some limitations. Our observation period was just 3 months and we only included patients diagnosed with POAG or OH. Moreover, the study design did not include any type of masking, which could have led to some bias in the results that were obtained. We also did not examine previous adherence status of our enrolled patients, which would have been interesting to compare before and after treatment switch. We plan to investigate this in future studies. Also, the patients in our study were using Xalatan drops prior to treatment switch and it would be of interest to assess outcomes for available generic formulations because they may differ in preservative or additives to the main active compound.

In conclusion, the results of this study suggest that a switch from latanoprost to tafluprost reduced the signs and symptoms of ocular surface conditions in patients with POAG and OH. Also, treatment with low preservative tafluprost demonstrated comparable IOP lowering effectiveness compared to latanoprost.
